# Partial genetic deletion of neuregulin 1 and adolescent stress interact to alter NMDA receptor binding in the medial prefrontal cortex

**DOI:** 10.3389/fnbeh.2014.00298

**Published:** 2014-09-29

**Authors:** Tariq W. Chohan, An Nguyen, Stephanie M. Todd, Maxwell R. Bennett, Paul Callaghan, Jonathon C. Arnold

**Affiliations:** ^1^The Brain and Mind Research Institute, University of SydneySydney, NSW, Australia; ^2^Discipline of Pharmacology, School of Medical Science, University of SydneySydney, NSW, Australia; ^3^ANSTO LifeSciences, Australian Nuclear Science and Technology OrganisationSydney, NSW, Australia

**Keywords:** schizophrenia, neuregulin 1, adolescence, stress, NMDA, medial prefrontal cortex, lateral septum, hippocampus

## Abstract

Schizophrenia is thought to arise due to a complex interaction between genetic and environmental factors during early neurodevelopment. We have recently shown that partial genetic deletion of the schizophrenia susceptibility gene neuregulin 1 (*Nrg1*) and adolescent stress interact to disturb sensorimotor gating, neuroendocrine activity and dendritic morphology in mice. Both stress and *Nrg1* may have converging effects upon N-methyl-D-aspartate receptors (NMDARs) which are implicated in the pathogenesis of schizophrenia, sensorimotor gating and dendritic spine plasticity. Using an identical repeated restraint stress paradigm to our previous study, here we determined NMDAR binding across various brain regions in adolescent *Nrg1* heterozygous (HET) and wild-type (WT) mice using [^3^H] MK-801 autoradiography. Repeated restraint stress increased NMDAR binding in the ventral part of the lateral septum (LSV) and the dentate gyrus (DG) of the hippocampus irrespective of genotype. Partial genetic deletion of *Nrg1* interacted with adolescent stress to promote an altered pattern of NMDAR binding in the infralimbic (IL) subregion of the medial prefrontal cortex. In the IL, whilst stress tended to increase NMDAR binding in WT mice, it decreased binding in *Nrg1* HET mice. However, in the DG, stress selectively increased the expression of NMDAR binding in *Nrg1* HET mice but not WT mice. These results demonstrate a Nrg1-stress interaction during adolescence on NMDAR binding in the medial prefrontal cortex.

## Introduction

Schizophrenia is thought to arise due to a complex interaction between genetic and environmental factors during critical early periods of neurodevelopment that result in disease onset in late adolescence/early adulthood (Weinberger, 1987; Murray et al., 1991; Lewis and Levitt, 2002; Van Winkel et al., 2008; Jaaro-Peled et al., 2009; Van Winkel et al., 2010; Van Os et al., 2010). Ionotropic N-methyl-D-aspartate receptors (NMDARs) mediate activity-dependent plasticity of glutamatergic synapses (Wenthold et al., 2003; Bennett, 2009) and play a key role in normal brain development through regulation of memory, attention and learning processes (Hudspith, 1997; Lieth et al., 2001; Bennett, 2009; Kantrowitz and Javitt, 2010).

Hypofunction of glutamatergic neurotransmission in the form of abnormal functioning of NMDARs in corticolimbic regions of the brain may explain the symptoms of schizophrenia (Carlsson and Carlsson, 1990; Bennett, 2009). For example, the administration of NMDAR antagonists such as phencyclidine (PCP) to humans induces most of the positive and negative symptom, as well as cognitive impairments observed in schizophrenia patients (Javitt, 1987). Similarly, administration of NMDAR antagonists like MK-801 in rodents, particularly during neurodevelopment, promotes lasting schizophrenia-relevant behavioral phenotypes such as locomotor hyperactivity, prepulse inhibition of startle (PPI) deficits, social withdrawal and cognitive dysfunction (Facchinetti et al., 1993; Sircar, 2000; Wang et al., 2001; Harris et al., 2003; Wiley et al., 2003; Andersen and Pouzet, 2004; Stefani and Moghaddam, 2005; Du Bois et al., 2008). Furthermore, post-mortem schizophrenia brain tissue studies have reported an increased binding of the radiolabelled NMDAR ligand MK-801 in the frontal cortex and caudate-putamen (Kornhuber et al., 1989; Newell et al., 2005). Although, reduced NMDAR sub-unit expression has recently been reported in schizophrenia brains which was accompanied by a reduced concentration of NMDA (Errico et al., 2013).

The neurotrophic factor neuregulin 1 (NRG1), is a widely accepted schizophrenia susceptibility gene which plays a significant role in normal brain maturation by influencing neuronal migration, myelination, and synaptic plasticity (Pearce et al., 1987; McDonald and Johnston, 1990; Stefansson et al., 2002; Harrison and Law, 2006; Mei and Xiong, 2008; Barros et al., 2009; Bennett, 2009, 2011). Interestingly, schizophrenia patients show altered expression of both the ErbB family of receptors for NRG1 and NMDARs (Stefansson et al., 2002; Chong et al., 2008; Alaerts et al., 2009; Hatzimanolis et al., 2013). The shared regulation of neuronal plasticity through the Nrg1-ErbB receptor and NMDARs systems has been demonstrated through an interaction in the post synaptic density (PSD) via the anchoring protein PSD-95 (Garcia et al., 2000; Huang et al., 2000; Bao et al., 2004; Murphy and Bielby-Clarke, 2008). Interestingly partial genetic deletion of *Nrg1* hypophosphorylates NR2B subunits of NMDARs (Bjarnadottir et al., 2007) and promotes subtle changes in NMDAR binding in a number of schizophrenia relevant brain regions in adult rodents (Dean et al., 2008; Long et al., 2013; Newell et al., 2013).

Schizophrenia etiology also consists of an environmental component. Early life stress might be the common denominator linking several environmental risk factors including urbanicity, cannabis use, migration, childhood trauma and obstetric complications (Geddes and Lawrie, 1995; Dalman, 2003; Myin-Germeys et al., 2003; Corfas et al., 2004; Glaser et al., 2006; Henquet et al., 2008; Walker et al., 2008; Van Os et al., 2010). Indeed, adolescence is a period of heightened risk to develop schizophrenia (Walker and Bollini, 2002; Costello et al., 2003; Paus et al., 2008). Increased stress reactivity during adolescence coincides with normal maturation of cognitive abilities, and rapid development of the prefrontal cortex (Leussis et al., 2008; Rahdar and Galvan, 2014) and stabilization of the hippocampus (Leussis et al., 2008). Both the prefrontal cortex and hippocampus are vulnerable to the negative effects of stress (Jinks and McGregor, 1997; Sullivan and Gratton, 1999; Buijs and Van Eden, 2000; McEwen, 2007). Moreover, these regions display schizophrenia brain pathology such as a reduced density of dendritic spines, small protrusions which support excitatory synapses in neuronal circuits (Weinberger and Lipska, 1995; Velakoulis et al., 1999; Eichenbaum, 2000; Glantz and Lewis, 2000; Preston et al., 2005; Von Bohlen Und Halbach et al., 2006; Lawrie et al., 2008; Ebdrup et al., 2010). Stress hormone exposure during adolescence in mice, alters the expression of NMDAR subunits in the prefrontal cortex and hippocampus (Lee et al., 2003; Sterlemann et al., 2010; Buret and Van Den Buuse, 2014), regions that regulate cognitive and sensorimotor gating, and are sensitive to stress-induced loss of dendritic spine density and gray matter losses (Kassem et al., 2013). No prior study has directly examined the effects of adolescent restraint stress on [^3^H] MK-801 binding in rodents. In adult rats chronic variable stress increased [^3^H] MK-801 binding in the prefrontal cortex and decreased binding in the hippocampus (Lei and Tejani-Butt, 2010). Given that both neuregulin and stress impact upon NMDARs in their own right, this opens the possibility that neuregulin might confer vulnerability to the effects of stress on NMDAR expression.

*Nrg1* confers vulnerability to the effects of environmental challenges of relevance to schizophrenia. Our laboratory has shown that partial genetic deletion of neuregulin 1 increases sensitivity to the neurobehavioral actions of cannabinoids (Boucher et al., 2007a,b, 2011; Long et al., 2010, 2012, 2013; Spencer et al., 2013) and methamphetamine (Spencer et al., 2012), both of which are drugs of abuse known to activate stress systems in the brain including the HPA axis (Gerra et al., 2003; Huizink et al., 2006; King et al., 2010; Van Leeuwen et al., 2011). We and others have also recently demonstrated that genetic variation in *Nrg1* confers vulnerability to the neurobehavioral effects of stress and modifies neuronal signaling pathways sub serving the stress response. For example, rats heterozygous for type II *Nrg1* display altered expression of glucocorticoid receptors in the pituitary, hippocampus and paraventricular nucleus of the hypothalamus (Taylor et al., 2010). Partial genetic deletion of *Nrg1* conferred vulnerability to the effects of adolescent social defeat stress on spatial working memory function and modulation of inflammatory cytokines in the prefrontal cortex and hippocampus (Desbonnet et al., 2012). We have also recently shown that partial genetic deletion of *Nrg1* altered neurobehavioral responses to repeated adolescent restraint stress (Chohan et al., 2014). Repeated adolescent stress selectively impaired the development of normal sensorimotor gating in *Nrg1* heterozygous (*Nrg1* HET) mice which correlated with a dysregulation in stress-induced corticosterone release. Furthermore, pyramidal neurons in the medial prefrontal cortex of *Nrg1* HET mice exposed to repeated adolescent restraint stress had shorter dendritic lengths and complexity, as well as an increased dendritic spine density. Here we hypothesize that repeated restraint stress, coupled with disrupted Nrg1-ErbB4 signaling during adolescence, might interact to alter NMDAR binding in the mouse brain.

## Methods

### Mice

Adolescent (PND 35-49) male *Nrg1* HET mice (C57BL/6JArc background strain) and wild-type (WT) littermates were used. The mice were bred in our animal house, sourced from a total of 9 litters and intermixed at weaning on postnatal day (PND) 21. Genotypes were determined after weaning at PND 21 as previously described (Karl et al., 2007). The mice were housed (4–5 animals per homecage) in a room on a 12:12 h light:dark reverse light schedule with food and water available *ad libitum*. Animals had access to environmental enrichment including a cardboard toilet roll, igloo, sunflower seeds, tissue paper and running wheels. Environmentally enriched housing is beneficial when exploring gene and environment interactions (G × E) in mice because it better approximates human cognitive and sensorimotor development than standard housing (Burrows et al., 2011). *Nrg1* HET mice were generated by Prof Richard Harvey (Victor Chang Cardiac Research Institute, Sydney) using a targeting vector in which most of exon 11, which encodes the transmembrane domain, was replaced by a neomycin resistance gene cassette (Stefansson et al., 2002). All research and animal care procedures were approved by the University of Sydney's Animal Ethics Committee and were in agreement with the Australian Code of Practice for the Care and use of Animals for Scientific Purposes.

### Experimental design

Male mice were subjected to 30 min/day of restraint stress for 14 days from PND 36 to PND 49 as described in our previous study (Chohan et al., 2014). Restraint stress was chosen as it is a well-characterized physical stressor in rodents that activates the HPA axis and increases anxiety-related behavior (Eiland and McEwen, 2010; Sutherland et al., 2010; Sutherland and Conti, 2011; Chesworth et al., 2012). Non-stressed animals (WT and *Nrg1* HET) did not receive restraint stress and remained undisturbed in their homecages, similar to prior methods (Eiland and McEwen, 2010; Eiland et al., 2012; Hill et al., 2013; Kwon et al., 2013). Stressed mice were placed in a restraint device (Harvard Apparatus, Holliston, MA, USA), which consisted of a close-ended clear perspex cylinder (9.5 × 2.5 cm). Mice were handled daily for 7 days prior to the commencement of experimentation and randomly allocated to 4 experimental groups: (1) WT-no stress (WT NS, *n* = 6); (2) WT-stress (WT S, *n* = 7); (3) *Nrg1* HET-no stress (*Nrg1* HET NS, *n* = 6), and (4) *Nrg1* HET-stress (*Nrg1* HET S, *n* = 5). Homecage controls and restraint stressed animals were sacrificed by cervical dislocation immediately following their final 30 min restraint stress episode on day 14 (PND 49) and their brains were extracted, snap frozen and stored at −80°C prior to sectioning.

### NMDA receptor autoradiography

**Figure 1:**
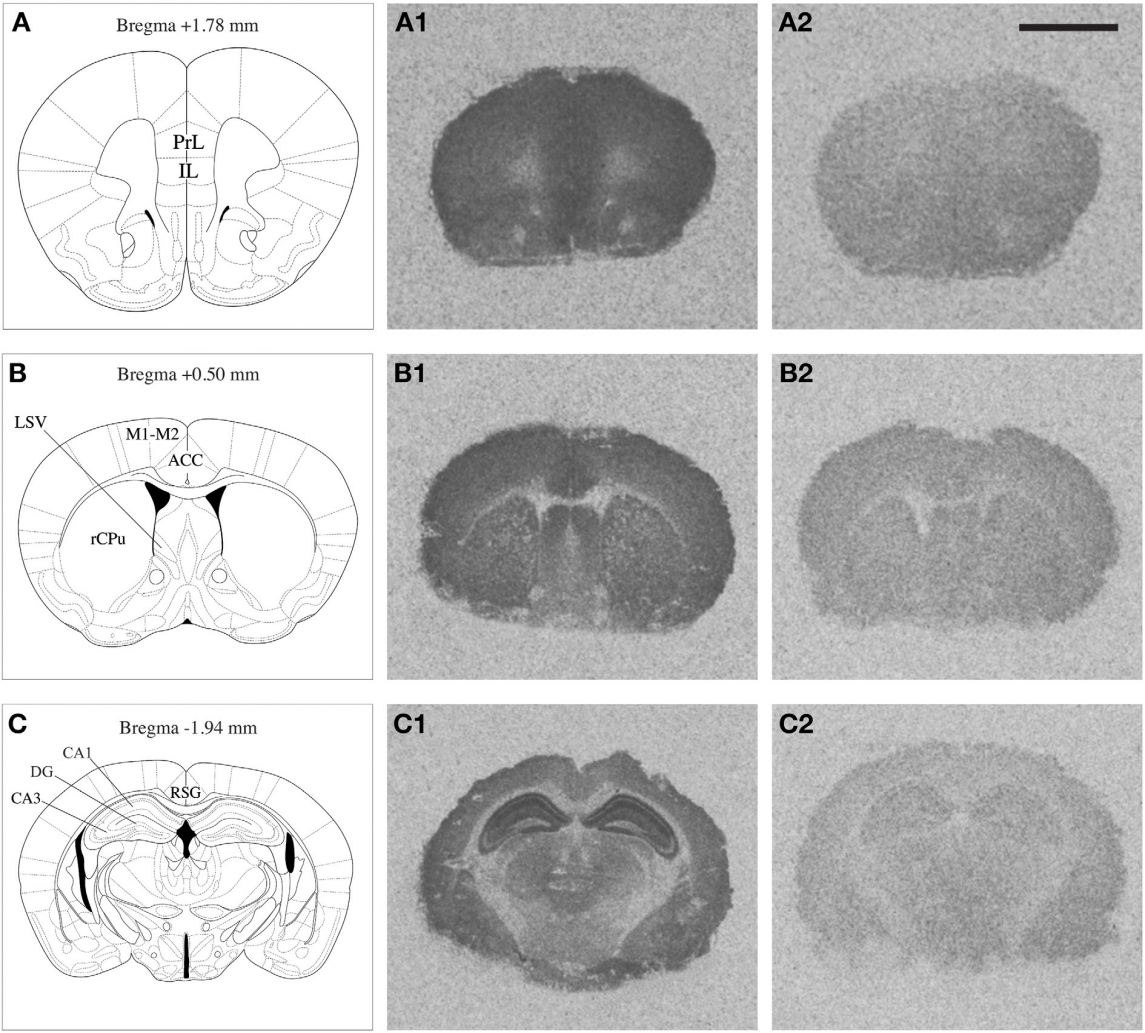
**Mouse brain atlas adapted from Paxinos (2004), indicating the brain regions quantified (A,B,C); PrL: prelimbic cortex, IL: infralimbic cortex, rCPu: rostral caudate putamen, M1-M2: motor cortex, ACC: anterior cingulate cortex, LSV: ventrolateral septum, RSG: retrosplenial granular cortex, DG: dentate gyrus, CA1 and CA3 subregions of the hippocampus**. Representative autoradiograms of coronal brain sections showing total [^3^H] MK-801 binding (A_1_,B_1_,C_1_) and non-specific [^3^H] MK-801 binding (A_2_,B_2_,C_2_). Scale bar = 2.5 mm.

NMDAR autoradiography was conducted on brains extracted from the same mice that were used to determine corticosterone levels reported previously by our research group (Chohan et al., 2014). In these mice a differential effect of repeated stress was observed between *Nrg1* HET and WT mice on plasma corticosterone concentrations. The whole brain was coronally sectioned at 20 μm on a cryostat, thaw-mounted onto polysine slides and stored at −80°C until use. Brain regions selected for quantification were identified based on a standard mouse brain atlas (Paxinos, 2004) at bregma levels +1.78 [containing prelimbic (PrL) and infralimbic (IL) cortices); +0.50 (containing anterior cingulate cortex (ACC), rostral caudate-putamen (rCPu), motor cortex (M1-M2), ventrolateral septum (LSV)]; and −1.94 (containing retrosplenial granular cortex (RSG), and subregions of the hippocampus including dentate gyrus (DG), CA1 (cornu ammonis area 1) and CA3 (cornu ammonis area 3) stratum radiatum layers (Figure 1). Our prior work showed that Nrg1 hypomorphism alone and in combination with stress affected dendritic morphology in the medial prefrontal cortex and hippocampus (Chohan et al., 2014) and so these regions were consequently analyzed for MK-801 binding in the present study. Furthermore, the medial prefrontal cortex and hippocampus are strongly implicated in the neurobiology of schizophrenia and stress (Michelsen et al., 2007; Radley et al., 2008; Alfarez et al., 2009). The caudal ACC region was examined, as it has been shown previously by our group to be affected by stress (Kassem et al., 2013) and is a point of comparison to another MK-801 binding study performed in *Nrg1* HET mice (Newell et al., 2013). Further, we examined the LSV at it is thought to mediate stress and anxiety-related behavior (Dielenberg et al., 2001; Sheehan et al., 2004) and was shown to be dysregulated in our prior work on Nrg1-cannabinoid interactions (Boucher et al., 2007b, 2011).

The sections were incubated in 30 mM HEPES buffer (pH 7.45) containing 23 nM [^3^H] MK-801 (specific activity 27.5 Ci/mmol, PerkinElmer, USA), 100 μM glycine, 100 μM L-glutamate and 1 mM EDTA for 2.5 h at room temperature. Non-specific binding was determined by incubating adjacent sections with [^3^H] MK-801 in the presence of 200 μM (ketamine hydrochloride, National Measurement Institute, Sydney, Australia). Following the incubation, the sections were washed twice for 20 min each at 4°C in 30 mM HEPES containing 1 mM EDTA (pH 7.45) and rapidly dried under a stream of cool air.

### Quantitative analysis of autoradiographic images

Following the binding assays, all sections were placed on Kodak BioMax MR Film along with a [^3^H] autoradiographic standard (Amersham, UK) for 4 or 6 weeks. Some of the samples from the CA1 region of the hippocampus were re-exposed for 4 weeks due to initial oversaturation of the films, to allow them to fall within the normal pseudolinear response range. Films were developed using Kodak GBX developer/fixer (Sigma-Aldrich, NSW, Australia). Films were scanned using a BioRad GS-800 calibrated densitometer, and quantification of mean density performed in each brain region [average optical density over three adjacent brain sections, for total binding and non-specific binding, using ImageJ (http://rsbweb.nih.gov/ij)]. Using density values for calibrated [^3^H] autoradiographic standards, radioactive concentrations were derived for all density values using a standard curve, and converted into fmol per mg tissue equivalent (fmol/mg). All regions quantified were analyzed blind to treatment group. Specific *in vitro* binding of [^3^H] MK-801 was calculated by subtraction of non-specific from total binding values. For each brain region, 6 frozen sections (3 total binding and 3 non-specific binding were selected per animal. Due to sectioning problems some of the sections were torn and unsuitable for processing. Therefore, for some of the brain regions the final value represents an average from five animals only.

### Statistical analyses

Statistical analyses were performed using SPSS (IBM, IL, USA) or Statview (SAS Institute Inc) software. Statistically significant variation in radioligand binding was identified by Two-Way analysis of variance (ANOVA) with genotype or stress as factors. Planned Bonferroni comparisons were conducted to further analyze differences between experimental groups on all measures using the following four specific comparisons (WT-no stress vs. *Nrg1* HET-no stress, WT-stress vs. *Nrg1* HET-stress, WT-no stress vs. WT-stress, and *Nrg1* HET-no stress vs. *Nrg1* HET stress). The results of all analyses were deemed significant at *p* < 0.05.

## Results

**Figure 2:**
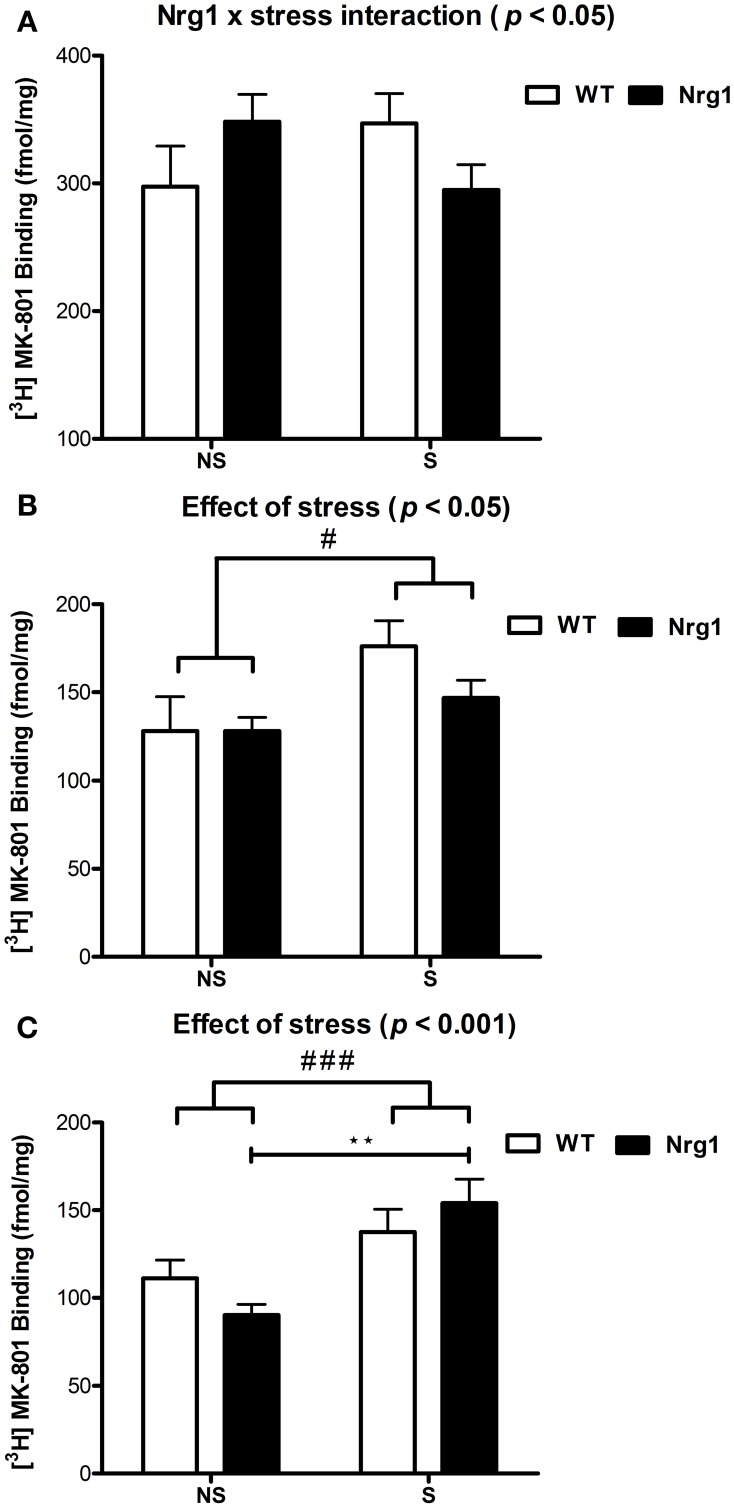
**[^3^H] MK-801 binding to NMDARs (fmol/mg tissue) in (A) infralimbic cortex (IL), (B) ventrolateral septum (LSV) and (C) dentate gyrus (DG) in repeatedly stressed (S) and non-stressed (NS) wild type-like (WT) and *Nrg1* HET mice; *n* = 5–7/group**. Data are presented as means ± s.e.m. Significant effects are indicated by #*p* < 0.05 and ###*p* < 0.001 (main effects of stress); ^**^p < 0.01 [planned Bonferroni comparison, *Nrg1* HET (NS) vs. *Nrg1* HET (S)].

In all groups, the highest density of specific binding was distinctly observed in the hippocampus (CA1 & CA3 subregions). Moderately high levels of [^3^H] MK-801 binding were observed in the PrL, IL and anterior cingulate cortices. The rCPu, RSG, M1-M2, DG and LSV subdivisions displayed moderate-low levels of [^3^H] MK-801 binding. Two factor ANOVA revealed a significant genotype by stress interaction (*F*_(1, 18)_ = 4.53, *p* < 0.05) in the IL cortex (Table 1, Figure 2A). A significant effect of stress was found for [^3^H] MK-801 binding in the LSV (*F*_(1, 19)_ = 5.58, *p* < 0.05) and DG (*F*_(1, 20)_ = 15.51, *p* < 0.001), demonstrating that restraint stress significantly increased NMDAR expression in these regions independent of genotype (Table 1 and Figure 2B). Planned Bonferroni comparisons revealed that stressed *Nrg1* HET mice exhibited greater MK-801 binding in the DG compared with their non-stressed counterparts (*p* < 0.01). There were no main effects of genotype or stress, or genotype by stress interactions for NMDAR binding in any other brain regions examined (Table 1). No significant NMDAR binding differences between specific experimental groups in the other brain regions were observed (*p* > 0.05).

**Table 1:** **Specific NMDAR binding densities in repeatedly stressed and non-stressed WT and *Nrg1* HET mice**.

**Brain**	**WT**				***Nrg1* HET**				**Two-Way ANOVA**					
**Regions**	**No Stress**		**Stress**		**No Stress**		**Stress**		**Genotype**		**Condition**		**G × E**	
	**Mean**	**s.e.m**.	**Mean**	**s.e.m**.	**Mean**	**s.e.m**.	**Mean**	**s.e.m**.	***F-value***	***p-value***	***F-value***	***p-value***	***F-value***	***p-value***
PrL	314.975	±26.642	342.732	± 22.813	349.642	±13.966	308.860	±23.018	*F*_(1, 18)_ = 0.0004	0.986	*F*_(1, 18)_ = 0.090	0.768	*F*_(1, 18)_ = 2.493	0.132
IL	297.697	±31.497	347.315	±23.179	348.422	±21.457	295.171	±19.492	*F*_(1, 18)_ = 0.001	0.977	*F*_(1, 18)_ = 0.006	0.941	*F*_(1, 18)_ = 4.527	**0.047**
rCPu	160.311	±18.842	200.519	±9.704	169.193	±11.588	160.594	±11.154	*F*_(1, 19)_ = 1.469	0.240	*F*_(1, 19)_ = 1.523	0.232	*F*_(1, 19)_ = 3.630	0.072
M1-M2	247.933	±16.090	264.826	±9.768	236.519	±11.668	224.474	±20.893	*F*_(1, 19)_ = 3.223	0.088	*F*_(1, 19)_ = 0.028	0.868	*F*_(1, 19)_ = 1.007	0.328
ACC	291.354	±26.510	324.255	±16.283	281.255	±11.494	262.916	±14.381	*F*_(1, 20)_ = 3.744	0.067	*F*_(1, 20)_ = 0.156	0.697	*F*_(1, 20)_ = 1.926	0.181
RSG	171.369	±11.506	169.808	±14.702	143.954	±9.942	184.995	±16.380	*F*_(1, 20)_ = 0.207	0.654	*F*_(1, 20)_ = 2.156	0.158	*F*_(1, 20)_ = 2.511	0.129
LSV	127.992	±19.497	176.069	±14.489	128.175	±7.725	146.736	±10.233	*F*_(1, 19)_ = 1.068	0.314	*F*_(1, 19)_ = 5.584	**0.029**	*F*_(1, 19)_ = 1.095	0.308
CA1	610.541	±27.062	619.457	±17.838	640.685	±23.267	678.384	±35.213	*F*_(1, 20)_ = 3.072	0.095	*F*_(1, 20)_ = 0.842	0.367	*F*_(1, 20)_ = 0.321	0.577
CA3	459.248	±42.928	458.411	±41.408	416.045	±23.161	443.388	±32.370	*F*_(1, 19)_ = 0.640	0.434	*F*_(1, 19)_ = 0.133	0.720	*F*_(1, 19)_ = 0.150	0.703
DG	111.176	±10.369	137.475	±13.169	90.114	±6.194	153.924	±13.829	*F*_(1, 20)_ = 0.041	0.842	*F*_(1, 20)_ = 15.510	**0.0008**	*F*_(1, 20)_ = 2.688	0.117

*Data expressed as mean fmol/mg tissue ± s.e.m. for all brain regions examined (n = 5–7/group). Significant values are highlighted in bold. PrL, prelimbic cortex; IL, infralimbic cortex; rCPu, rostral caudate putamen; M1-M2, motor cortex; ACC, anterior cingulate cortex; LSV, ventrolateral septum; RSG, retrosplenial granular cortex; DG, dentate gyrus; CA1 and CA3 subregions of the hippocampus*.

## Discussion

Here we show that in adolescence partial genetic deletion of *Nrg1* promoted an idiosyncratic change in medial prefrontal cortex NMDAR binding in response to repeated stress. Repeated stress exposure tended to decrease [^3^H] MK-801 binding in *Nrg1* HET mice whilst promoting an increase in binding in WT mice in the IL cortex, a subregion of the medial prefrontal cortex. In the DG region of the hippocampus, stress significantly increased NMDAR binding. Interestingly, stressed *Nrg1* HET mice displayed significantly higher NMDAR binding than non-stressed *Nrg1* HET mice, an effect that was absent in WT mice. In addition, we report for the first time that restraint stress increased [^3^H] MK-801 binding levels in the LSV.

Partial genetic deletion of *Nrg1* failed to significantly alter NMDAR binding in the other brain regions examined (PrL, rCPu, RSG, ACC, motor cortex & CA1/CA3 regions of the hippocampus) when measured in late adolescence (PND 49). Prior studies have shown that adult *Nrg1* HET mice (> PND 60) display unaltered [^3^H] MK-801 binding in the cortex, caudate putamen, hippocampus and the septum (Dean et al., 2008; Long et al., 2013). Inconsistent with our present findings in late adolescent mice, adult *Nrg1* HET mice exhibited increased NMDAR binding in the ACC and motor cortex compared to WT mice (Newell et al., 2013). The differences observed between the current study and the findings of Newell et al. (2013) may be explained by the different developmental period examined between the studies. As NMDARs undergo significant changes across development (Scheetz and Constantine-Paton, 1994; Cull-Candy et al., 2001; Haberny et al., 2002) and the locomotor hyperactivity phenotype of *Nrg1* HET mice develops over time (Karl et al., 2007), it is possible that the effect of partial genetic deletion of *Nrg1* on NMDAR binding might also follow a developmental trajectory and become significant in adulthood.

Here we show for the first time that repeated restraint stress in adolescence increased NMDAR binding in the LSV. The LSV is responsible for promoting active behavioral responses in stressful situations (De Oca and Fanselow, 2004; Sheehan et al., 2004) and its ablation provoked septal rage and exaggerated defensive behaviors (Brady and Nauta, 1953). These findings imply that the integrity of the lateral septum is vital for the inhibition of excessive fear and anxiety. New evidence however indicates that the role of the lateral septum in controlling fear and anxiety is more complex than this, as infusion of CRF type 2 receptor agonists or optogenetic transient activation of CRF type 2 receptors in the lateral septum promoted anxiety-related behaviors (Henry et al., 2006; Anthony et al., 2014). Little research has examined the role of NMDARs in the lateral septum in the control of defensive behaviors. NMDAR knockout mice display reduced aggressive behavior and swim-stress induced Fos expression in the lateral septum than WT mice (Duncan et al., 2009).

The ability to directly compare studies examining the effects of stress on NMDARs is limited by factors including the diversity of stress paradigms implemented, differences in post-stress washout periods, and the multitude of methods used to analyse NMDAR expression. Most studies have explored the effect of stress on NMDAR subunit protein & mRNA expression, rather than total NMDAR binding as measured with [^3^H] MK-801 autoradiography (Sterlemann et al., 2010; Buret and Van Den Buuse, 2014). No prior study has directly examined the effects of adolescent restraint stress on [^3^H] MK-801 binding in rodents. In adult rats chronic variable stress increased [^3^H] MK-801 binding in the prefrontal cortex, caudate putamen, nucleus accumbens and basolateral amygdala, while decreasing binding in the hippocampus (Lei and Tejani-Butt, 2010). Here we could only discern measurable effects of stress on the LSV and DG, which might be explained by our use of a relatively mild restraint stress paradigm (30 min per day for 14 days). To resolve the effects of stress on NMDAR binding in other brain regions might require a more intense stress regimen like the classic paradigm of 6 h per day for 21 days that reliably induces retraction of dendrites and loss of gray matter (Radley et al., 2004, 2006, 2008; Magarinos et al., 2011; Kassem et al., 2013). Alternatively, it is possible that earlier application of the stressor (i.e., from PND 28) might have been more effective as recent data suggests that peripubertal stressor exposure (i.e., encompassing the juvenile through to pubertal period, PND 28-42 in rats) is critical to provoking neurobiological changes in stress circuits including increased NMDAR expression (Tzanoulinou et al., 2014).

Using an identical adolescent stress protocol we recently reported that partial genetic deletion of *Nrg1* and repeated stress interacted to offset the normal development of sensorimotor gating and blunted stress-induced corticosterone levels (Chohan et al., 2014). We also provided evidence of abnormal dendritic morphology in the medial prefrontal cortex of *Nrg1* HET mice exposed to stress. Specifically, unlike WT mice whose dendritic morphology was unaffected by stress, repeated stress in *Nrg1* HET mice reduced the length of dendrites and their complexity, and promoted an increase in dendritic spine density in pyramidal neurons of layers II/III of the anterior cingulate and PrL cortices of the medial prefrontal cortex. Given that Nrg1 and stress both influence NMDARs (Garcia et al., 2000; Bjarnadottir et al., 2007; Law et al., 2007; Li et al., 2007; Chong et al., 2008; Bennett, 2009; Cohen et al., 2010; Bennett et al., 2011; Buret and Van Den Buuse, 2014) and that NMDARs regulate the density of dendritic spines (Alvarez et al., 2007; Hayashi-Takagi et al., 2010) we hypothesized that Nrg1 and stress might interact to alter NMDAR binding specifically in the anterior cingulate and PrL cortices.

Therefore, it was surprising to observe in the present study that the Nrg1-stress interaction on NMDAR binding occurred in the IL cortex rather than the PrL cortex. The IL cortex shares reciprocal connections with the PrL cortex (Gabbott et al., 2003, 2005; Jones et al., 2005; Hoover and Vertes, 2007; Gutman et al., 2012) and the IL and PrL regions of the medial prefrontal cortex cooperate to produce an integrated response to stress (McDougall et al., 2004). Therefore, it is possible then that the changes in dendritic morphology in the anterior cingulate and PrL cortices in our previous study (Chohan et al., 2014) may be a cause or consequence of the Nrg1-stress interaction on NMDAR binding in the IL cortex we observed here. Indeed, perturbation of activity in the IL has flow on effects on the PrL cortex, as activation of IL cortex output via optical stimulation in adult rats inhibits PrL pyramidal neurons (Ji and Neugebauer, 2012). Here, there was a tendency toward reduced [^3^H] MK-801 binding in the medial prefrontal cortex of *Nrg1* HET mice which accords with the general view of NMDAR hypofunction in schizophrenia as well as research showing that NMDAR expression is reduced in the schizophrenia brain (Errico et al., 2013). Although, this contradicts studies that report [^3^H] increased MK-801 binding in post-mortem schizophrenia brains (Kornhuber et al., 1989; Newell et al., 2005).

Here we report that repeated stress-induced increased NMDAR binding in the DG in *Nrg1* HET mice but not in WT mice, which provides some additional support for *Nrg1* HET mice being more sensitive to the effects of stress on NMDAR binding. However, this must be interpreted cautiously in the absence of an overall interaction between Nrg1 genotype and stress condition. The DG plays an important role in memory and sensorimotor gating function (Reul et al., 2009; Guo et al., 2013), thus the stress induced increase in NMDAR binding specifically in *Nrg1* HET mice observed here may partially explain the spatial memory and PPI deficits observed previously in these mice following adolescent stress (Desbonnet et al., 2012; Chohan et al., 2014). Juvenile stress decreases expression of type III Nrg1 in the hippocampus (Brydges et al., 2014), so it is possible that the effects of stress on an already depleted Nrg1 level in hypomorphic mice is sufficient to then increase [^3^H] MK-801 binding. Why the DG but not the CA1 or CA3 region is selectively vulnerable to this effect is unclear. It might be partially explained by the DG expressing relatively lower levels of NRG1 than other hippocampal subfields (Law et al., 2004). The mechanisms responsible for the effect of stress on [^3^H] MK-801 binding in *Nrg1* HET mice will need to be specifically addressed in future research including studies which directly examine the expression, internalization and phosphorylation status of NMDAR, and also whether this effect can be magnified by a more intense stress protocol.

Our findings further reinforce research showing that variation in *Nrg1* confers vulnerability to the effects of stress. Human studies have shown that a *NRG1* polymorphism interacted with psychosocial stress to effect reactivity to expressed emotions in schizophrenia patients (Keri et al., 2009) and that polymorphic variation in *NRG1* interacts with job strain to increase the risk of heart disease (Hintsanen et al., 2007). Reduced type II *Nrg1* expression in rats induced increased baseline corticosterone levels, a disruption in recovery of stress-induced plasma corticosterone concentrations, as well as elevated levels of glucocorticoid receptors in the hippocampus, paraventricular nucleus of the hypothalamus and pituitary gland (Taylor et al., 2010). Further, complex gender specific interactions of type II *Nrg1* genotype and adolescent chronic variable stress were reported on anxiety-related behavior and cued fear conditioning (Taylor et al., 2012). Stress-induced increase in corticosterone was more pronounced in *Nrg1* HET mice than WT mice at the younger (3–4 months) but not the older age group (6–7 months) (Chesworth et al., 2012), highlighting the developmental effect of stress and Nrg1 hypomorphism on the HPA axis. Adolescent social defeat stress has also been shown to selectively impair spatial memory and decrease expression of the inflammatory cytokine interleukin 1β in the prefrontal cortex of *Nrg1* HET mice, but not WT mice (Desbonnet et al., 2012). The latter finding might be related to the present finding of partial genetic deletion of *Nrg1* promoting a unique stress-induced downregulation of NMDAR binding in the prefrontal cortex, as interleukin 1β (IL 1β) has been shown to potentiate NMDA function and reduce the density of synaptic spines (Viviani et al., 2003, 2006). Further, the effects of IL 1β are mediated by interleukin receptor 1 (ILR1) which appear to interact with NR2B subunits of the NMDAR in the postsynaptic density (Gardoni et al., 2011; Viviani et al., 2013).

### Conflict of interest statement

The authors declare that the research was conducted in the absence of any commercial or financial relationships that could be construed as a potential conflict of interest.
